# Advances in Insulin Delivery: Transdermal and Needle-Free Technologies as Emerging Strategies to Improve Metabolic Control and Treatment Adherence

**DOI:** 10.3390/life16030377

**Published:** 2026-02-26

**Authors:** Manuel García-Sáenz, Oscar Josué Gómez-Romero, Etual Espinosa-Cárdenas, Claudia Ramírez-Rentería, José Luis Eduardo Doval-Caballero, Daniel Uribe-Cortés, Aldo Ferreira-Hermosillo

**Affiliations:** 1 Servicio de Endocrinología, Hospital de Especialidades del Centro Médico Nacional Siglo XXI, Instituto Mexicano del Seguro Social, Mexico City 06720, Mexico; manuel.garcias@unam.edu (M.G.-S.); oscar25grtxc@gmail.com (O.J.G.-R.); eespinosa@endocrinologiamx.org (E.E.-C.); 2 Unidad de Investigación Médica en Enfermedades Endocrinas, Centro Médico Nacional Siglo XXI, Instituto Mexicano del Seguro Social, Mexico City 06720, Mexico; cramirez@endocrinologia.org.mx; 3 Clínica de Obesidad, Instituto Nacional de Ciencias Médicas y Nutrición “Salvador Zubirán”, Mexico City 14080, Mexico; eduardo.dovalc@incmnsz.mx; 4 Hospital Ángeles Clínica Londres Torre Frontera, Mexico City 06700, Mexico; dr.daniel.uc@gmail.com

**Keywords:** insulin delivery, transdermal insulin, needle-free injection, jet injector, microneedles, metabolic control, glycemic variability, treatment adherence, diabetes mellitus

## Abstract

Insulin therapy remains essential for the management of diabetes mellitus; however, conventional subcutaneous injection continues to impose significant physical, psychological, and behavioral barriers that negatively affect treatment adherence and metabolic outcomes. Injection-related pain, fear of needles, local tissue complications, and psychological insulin resistance contribute to delayed insulin initiation, inadequate dose titration, and suboptimal glycemic control worldwide. In response, alternative insulin delivery routes (including oral, pulmonary, nasal, and transdermal strategies) have been explored to reduce invasiveness and improve patient experience. Among these, transdermal insulin delivery has emerged as a particularly promising approach due to its potential to bypass gastrointestinal degradation, provide controlled absorption, and enhance patient acceptance. Recent advances in microneedle-based systems and needle-free jet injectors have enabled effective transdermal insulin administration by overcoming the skin barrier while minimizing pain and discomfort. This narrative review synthesizes current evidence on insulin delivery technologies with a specific focus on transdermal and needle-free systems. We discuss the biological and physicochemical challenges of insulin transport, the mechanisms underlying emerging delivery platforms, and clinical evidence regarding metabolic efficacy, glycemic variability, and patient-reported outcomes. The integration of these technologies with continuous glucose monitoring is also explored. Finally, we address translational challenges and future perspectives, highlighting the role of needle-free insulin delivery as a patient-centered strategy to improve adherence and metabolic control in diabetes care.

## 1. Introduction

Diabetes mellitus is a heterogeneous group of metabolic disorders characterized by chronic hyperglycemia resulting from impaired insulin secretion, insulin action, or both. The conditions are associated with profound alterations in carbohydrate, lipid, and protein metabolism and lead to progressive microvascular and macrovascular complications when glycemic control is suboptimal. Despite significant advances in pharmacological therapies and glucose monitoring technologies, insulin remains indispensable for individuals with type 1 diabetes and for a substantial proportion of those with type 2 diabetes [1,2,3].

However, achieving optimal metabolic control extends far beyond the molecular characteristics of insulin formulations. The route of administration, device usability, and patient perception of therapy play critical roles in long-term adherence and treatment success. Conventional subcutaneous insulin injection, although effective, is frequently associated with pain, discomfort, lipohypertrophy, and fear of needles. These factors contribute to psychological insulin resistance, delayed initiation of insulin therapy, and inadequate dose adjustment, ultimately compromising glycemic outcomes [3,4,5,6].

Over the past decades, substantial efforts have been made to improve insulin pharmacokinetics through molecular modifications and the development of rapid-acting and long-acting analogues. In parallel, delivery devices have evolved from syringes to insulin pens, pumps, and patch-based systems. However, even with these advances, the need for repeated needle-based injections remains a major limitation. Consequently, there has been growing interest in alternative insulin delivery routes that minimize invasiveness while maintaining therapeutic efficacy [6,7,8].

Beyond behavioral barriers, subcutaneous insulin delivery exhibits inherent pharmacokinetic variability. Factors such as injection depth, local blood flow, tissue composition, and lipohypertrophic areas influence insulin dispersion and absorption. Even rapid-acting analogues display variability in time-to-peak concentration and early postprandial action, which may translate into glycemic excursions and increased intra-individual glucose variability [7,9].

In this context, innovation in insulin delivery systems has progressively shifted from purely pharmacological optimization toward device- and route-based solutions. Transdermal strategies, microneedle platforms, and needle-free jet injectors aim not only to improve patient comfort but also to modify absorption kinetics, enhance metabolic predictability, and potentially reduce glycemic variability through more consistent insulin dispersion. Importantly, recent advances in polymer-mediated skin permeation and ionic liquid-based systems suggest that the long-standing barrier imposed by the skin may be mechanistically modifiable [10,11].

Among the explored strategies, transdermal insulin delivery has gained increasing attention due to its potential to combine metabolic effectiveness with improved patient comfort. Microneedle-based systems and needle-free jet injectors represent innovative technologies capable of overcoming the skin barrier while reducing pain perception and improving treatment acceptance. Emerging quantitative data from preclinical and early clinical studies now allow a more critical evaluation of their pharmacokinetic performance and translational feasibility [8,12,13,14,15].

This review aims to contextualize these advances within the broader landscape of insulin delivery technologies and to highlight their relevance for metabolic control and to critically examine their mechanistic basis, clinical performance, and real-world applicability for improving control and adherence.

## 2. Global Burden of Diabetes and Challenges of Insulin Therapy

Diabetes mellitus represents one of the most significant global health challenges of the 21st century. Its prevalence continues to rise, driven by population aging, urbanization, sedentary lifestyles, and increasing rates of obesity. The metabolic complexity of diabetes necessitates continuous medical care and active patient self-management to achieve glycemic targets and prevent long-term complications [5,6].

Insulin therapy is often perceived by patients as a marker of disease progression or treatment failure, particularly in type 2 diabetes. This perception, combined with fear of injections and concerns about hypoglycemia, contributes to resistance against insulin initiation and intensification. Multiple daily injections require technical training, frequent glucose monitoring, and ongoing dose adjustments, which impose a considerable burden on patients and healthcare systems alike [6].

Furthermore, repeated subcutaneous injections are associated with local adverse effects such as bruising, pain, inflammation, infection, and lipodystrophy. These complications can alter insulin absorption and further increase glycemic variability. From a behavioral perspective, injection-related discomfort and inconvenience are strongly associated with poor adherence, missed doses, and inconsistent treatment patterns, all of which negatively impact metabolic outcomes [16,17].

Consequently, improving insulin delivery methods represents a critical unmet need in diabetes care. An ideal delivery system should not only provide reliable insulin absorption but also reduce pharmacokinetic variability, enable flexible dose titration, minimize discomfort, and facilitate integration with contemporary technologies such as continuous glucose monitoring [5,18].

## 3. Overview of Insulin Delivery Routes

### 3.1. Conventional Subcutaneous Administration

Subcutaneous injection remains the standard route for insulin delivery due to its reliability and predictable pharmacokinetics. However, absorption from subcutaneous tissue can be influenced by external factors, leading to variability in insulin action. This variability is particularly relevant in the early postprandial phase, where differences in dispersion and tissue perfusion may delay peak insulin activity and contribute to glycemic excursions [7,9].

Despite improvements in needle design and injection devices, pain and needle aversion remain common.

### 3.2. Oral Insulin Delivery

Oral insulin delivery has long been pursued to replicate physiological portal insulin exposure. However, enzymatic degradation in the gastrointestinal tract and poor intestinal permeability severely limit bioavailability. Although advanced formulation strategies such as nanoparticles and polymer-based carriers have shown promise, oral insulin remains largely experimental [7,8,14,19].

### 3.3. Pulmonary Insulin Delivery

Pulmonary insulin exploits the large alveolar surface area for rapid systemic absorption. While effective in reducing postprandial hyperglycemia, its clinical use has been limited by concerns regarding pulmonary safety, dosing variability, and restricted patient eligibility [7,8,14].

### 3.4. Nasal Insulin Delivery

Intranasal insulin provides rapid absorption through the nasal mucosa but is hampered by low permeability, mucosal irritation, and inconsistent pharmacokinetics, limiting its long-term clinical applicability [7,8,14].

### 3.5. Rationale for Transdermal Insulin Delivery

Transdermal delivery offers several theoretical advantages over other non-invasive routes, including avoidance of gastrointestinal degradation, potential for sustained insulin release, and improved patient comfort. The primary obstacle to this approach is the skin itself, which constitutes a highly effective barrier to macromolecules such as insulin. Overcoming this barrier in a safe and acceptable manner has been the focus of significant technological innovation [9,14,15].

Importantly, successful transdermal insulin systems must demonstrate not only enhanced permeation but also clinically meaningful glucose reduction, dose scalability, reproducibility of absorption, and acceptable long-term dermatologic safety. These translational criteria remain central when interpreting recent advances in chemical permeation enhancers, microneedle systems, and polymer-mediated transport platforms [14].

## 4. The Skin as a Biological Barrier to Insulin Delivery

The skin constitutes one of the most efficient protective barriers in human physiology. Its primary function is to prevent excessive water loss and protect against external chemical, microbial, and mechanical insults. From a drug delivery perspective, however, this protective role represents a major obstacle, particularly for large and hydrophilic molecules such as insulin [9].

The outermost layer of the skin, the stratum corneum, is composed of corneocytes embedded in a highly organized lipid matrix, often described as a “brick-and-mortar” structure. This architecture restricts passive diffusion to small, lipophilic molecules with molecular weights typically below 500 Da. Insulin, with a molecular weight of approximately 5700 Da and a hydrophilic protein structure, is therefore virtually impermeable to intact skin under normal conditions [14].

Below the stratum corneum, the viable epidermis and dermis are richly vascularized and would allow efficient systemic absorption if insulin could reach these layers. Importantly, experimental evidence suggests that the viable epidermis itself may constitute an additional rate-limiting barrier, not merely the stratum corneum further complicating transdermal delivery strategies [9].

Consequently, most transdermal insulin delivery strategies aim to temporarily disrupt or bypass the stratum corneum without causing permanent skin damage or unacceptable discomfort. Over the past decades, multiple approaches have been investigated to overcome this barrier. These strategies can be broadly classified into chemical, electrical, mechanical, and microstructural methods, each with distinct mechanisms, advantages, and limitations [9,15].

## 5. Strategies to Overcome the Skin Barrier for Transdermal Insulin Delivery

The skin, particularly the stratum corneum, represents a highly efficient barrier to macromolecular transport. Insulin, with a molecular weight of approximately 5.8 kDa and hydrophilic characteristics, does not passively permeate intact skin at clinically meaningful levels. Historically, most enhancement strategies focused on disrupting SC lipid organization. However, emerging evidence indicates that deeper epidermal structures also constitute significant barriers, requiring more sophisticated permeation strategies [7,13,14].

**Figure 1:**
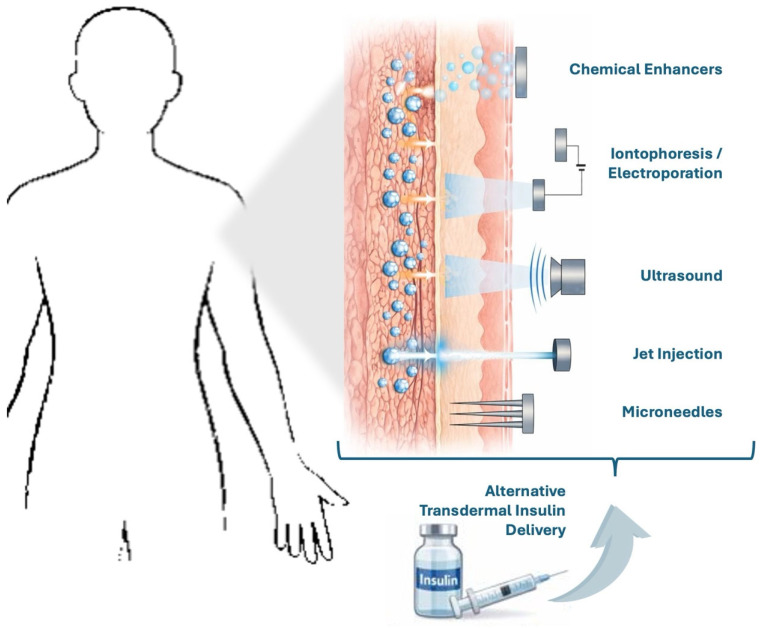
Schematic representation of transdermal insulin delivery strategies across the skin barrier. Chemical penetration enhancers, iontophoresis/electroporation, ultrasound-assisted delivery, needle-free jet injection, and microneedle-based systems facilitate insulin transport across the epidermis and dermis through distinct physical and biochemical mechanisms. Adapted from Zhang, et al. [14].

An overview of the main transdermal insulin delivery approaches and their mechanisms of action across the skin layers is illustrated in Figure 1.

### 5.1. Chemical Penetration Enhancers

Chemical penetration enhancers act by altering the lipid organization of the stratum corneum, increasing its permeability. Commonly studied agents include surfactants, fatty acids, alcohols, and solvents. While these compounds can enhance transdermal flux, their application in insulin delivery is limited by several factors [7,9,15].

First, the degree of permeability enhancement is often insufficient to achieve therapeutically relevant insulin concentrations. Second, chemical enhancers may destabilize insulin’s tertiary structure, reducing bioactivity. Third, repeated exposure can lead to skin irritation, inflammation, or barrier disruption, compromising long-term safety [20].

Importantly, most conventional chemical enhancers increase permeability by only 2–10-fold, which remains insufficient for macromolecules such as insulin unless combined with additional physical or structural modifications. For these reasons, chemical enhancers alone have not proven adequate for clinical insulin delivery [7,9,15].

### 5.2. Energy-Based Enhancement Techniques

Electrical and acoustic methods have been explored to transiently increase skin permeability:

○ Iontophoresis applies a low-intensity electrical current to drive charged molecules across the skin.
○ Electroporation uses short, high-voltage pulses to create transient aqueous pores in the stratum corneum.
○ Ultrasound (sonophoresis) increases permeability through cavitation and localized hyperthermia.

Although these methods can enhance insulin transport, their clinical application is limited by device complexity, discomfort, inconsistent dosing, and concerns regarding tissue damage. As a result, they are largely experimental and have not been widely adopted in routine diabetes care [7,14].

Experimental studies have reported transient increases in skin permeability ranging from 5 to 100-fold depending on voltage intensity or acoustic energy; however, reproducibility of insulin plasma levels and precise dose control remain inconsistent across studies [19].

### 5.3. Mechanical and Forced-Transport Approaches

Mechanical strategies physically force insulin across the skin barrier. These include ultrasound-assisted delivery and high-pressure liquid jet injection, which propels insulin through the skin using kinetic energy rather than a solid needle. Among mechanical methods, jet injection has shown the greatest translational potential and is discussed in detail below [7,9].

Unlike purely chemical enhancement strategies, mechanical approaches do not rely on altering insulin chemistry or lipid fluidization but instead bypass the barrier through physical force or microstructural disruption, thereby preserving the pharmacological properties of conventional insulin formulations [21].

### 5.4. Microneedle-Based Systems

Microneedle platforms represent an intermediate strategy between purely chemical transdermal systems and conventional subcutaneous injection. Rather than relying on lipid fluidization or macromolecular conjugation, microneedles create micron-scale channels that physically bypass the stratum corneum while minimizing nociceptor stimulation. This approach preserves the pharmacological integrity of insulin while reducing injection-associated discomfort [7,14].

Microneedle systems can be classified as:

○ Solid microneedles, which create microchannels for subsequent drug diffusion.
○ Hollow microneedles, which allow direct infusion of insulin.
○ Dissolving or biodegradable microneedles, composed of polymers that encapsulate insulin and dissolve within the skin.
○ Hydrogel-forming microneedles, which swell upon insertion and act as controlled-release reservoirs.

From a mechanistic standpoint, microneedles reduce diffusion distance and eliminate the lipid barrier imposed by the stratum corneum, thereby enabling near-physiologic absorption kinetics [22].

Preclinical and early clinical studies have demonstrated that microneedles can achieve rapid insulin absorption with favorable pharmacokinetics and high patient acceptability. Notably, together with needle-free jet injectors, microneedle systems represent the transdermal and minimally invasive technologies with the most human data available to date, although these studies remain limited in size and duration. However, large randomized controlled trials in populations with diabetes remain limited, and most data derive from preclinical models or small pilot studies [5,15].

Advanced glucose-responsive microneedle systems incorporating phenylboronic acid or enzymatic glucose oxidase triggers have demonstrated closed-loop potential in murine and minipig models, maintaining normoglycemia for extended periods. Nevertheless, translation to clinical practice remains constrained by manufacturing complexity and regulatory considerations [22].

A critical limitation of microneedle systems relates to payload capacity. Typical dissolvable patches measuring 1–2 cm^2^ can accommodate approximately 1–2 mg of insulin. While sufficient for low-dose basal or prandial needs in some patients, this may be inadequate for individuals requiring higher bolus doses (>15 IU), particularly in insulin-resistant populations. Scaling patch surface area increases manufacturing demands and may affect mechanical integrity [14,15,22].

Furthermore, real-time dose titration presents a challenge. Unlike pen or jet systems that allow incremental adjustments, fixed-dose microneedle patches may require redesign or multiple patch applications to achieve dose flexibility. This constraint could limit adaptability in patients requiring frequent titration [15].

From a conceptual perspective, microneedles offer a compelling compromise: they maintain conventional insulin dosing without the supraphysiologic escalation observed in chemical permeation strategies, yet they reduce pain perception compared to traditional needles. However, their widespread adoption will depend on scalable manufacturing, sterility assurance, and demonstration of long-term safety in diverse patient populations [12,14,15,22].

### 5.5. Microdermabrasion and Experimental Barrier Removal

Beyond microneedle-mediated microchannel formation, experimental models have explored more extensive physical removal of the stratum corneum and superficial epidermis as a means of enhancing insulin permeation. These approaches provide important mechanistic insights into the relative contribution of different skin layers to macromolecular transport and help clarify whether superficial lipid disruption alone is sufficient for clinically meaningful systemic absorption [22].

The assumption that the stratum corneum alone constitutes the dominant barrier has been challenged by Andrews et al. Their study demonstrated that removal of the SC alone did not significantly reduce blood glucose levels. Only removal of the full epidermis enabled insulin absorption sufficient to produce glycemic reduction comparable to 0.1 IU subcutaneous injection (approximately 0.35 IU/kg) [23].

Moreover, microdermabrasion produced a larger Cmax and greater area-under-curve (AUC) compared with injection, indicating enhanced system exposure. These findings implicate the viable epidermis and possibly the basal lamina and tight junctions as additional rate-limiting barriers [23].

This observation further supports the notion that strategies targeting only superficial lipid disruption may be insufficient for clinically meaningful insulin delivery, and that full barrier bypass may be required for predictable systemic absorption [23].

## 6. Emerging Chemical–Mechanical Hybrid Approaches

While microneedles and jet injectors rely primarily on physical barrier bypass, recent developments in ionic liquid systems and polymer-mediated transport attempt to chemically reprogram the permeability of the stratum corneum. These strategies differ fundamentally in their requirement for supraphysiologic dosing and chemical modification of insulin, raising important translational considerations [11,20].

### 6.1. Ionic and Lipid-Based Chemical Systems

In contrast to microneedle-based and jet injection systems, which primarily rely on physical barrier bypass, a second group of emerging strategies seeks to chemically modulate or reprogram the permeability of the stratum corneum. These approaches attempt to enhance insulin transport through lipid matrix disruption, nanocarrier-mediated intercellular diffusion, or polymer-assisted molecular transversal without the need for microchannel formation. While mechanistically distinct, these systems share a common translational challenge: achieving therapeutically relevant systemic insulin levels without requiring supraphysiologic dosing. The principal chemical and hybrid permeation strategies are discussed below [11,12,14].

#### 6.1.1. Ionic Liquid-in-Oil Systems

A recent development in this field involves ionic liquid-in-oil (IL/O) patch systems. Li et al. [10] engineered a microemulsion-based transdermal patch incorporating choline oleate and choline propionate within an adhesive matrix. Mechanistically, the IL/O formulation increases lipid fluidity within the stratum corneum and enhances intercellular transport [10,20].

In diabetic murine models, a subcutaneous dose of 10 IU/kg served as comparator. In contrast, the IL/O patch required 50 IU/kg to achieve sustained glycemic control, maintaining stable blood glucose levels for up to 72 h [20].

This fivefold increase in required dose highlights a recurrent challenge in chemical permeation systems: while sustained exposure may be achieved, efficiency of transport remains significantly lower than conventional injection, raising concerns regarding cost, insulin stability at high concentrations, and scalability [20].

#### 6.1.2. Polyzwitterion-Mediated Permeation

Wei et al. (2025) introduced a fast skin-permeable polyzwitterion polymer capable of conjugating with insulin and facilitating non-invasive transdermal delivery [11].

Quantitatively, topical administration of 116 IU/kg OP-conjugated insulin reduced blood glucose from approximately 400 mg/dL to below 200 mg/dL within one hour and maintained normoglycemia for roughly 12 h. Plasma insulin peaked near 230 μU/mL at one hour and remained 1.6–6 times higher than subcutaneous injection beyond two hours [11].

Although mechanistically elegant, the approximately 20-fold higher dose compared to conventional subcutaneous administration (~5 IU/kg in comparable models) underscores the persistent efficiency gap between chemical transdermal permeation and mechanical bypass approaches [11].

#### 6.1.3. Ethosome and Trans-Ethosome Nanovesicles

Ethosome and trans-ethosome systems further illustrate lipid-mediated enhancement strategies. In diabetic mice, ET formulations reduced blood glucose by 62% and maintained reductions for over 15 h, compared to approximately two hours with injection. However, dosing required 30 IU/kg—again exceeding typical subcutaneous equivalents [20].

These systems demonstrate enhanced duration of action, potentially favoring basal-like delivery patterns. Yet the need for dose escalation remains a consistent theme across chemical enhancement strategies [20].

A comparative overview of the major transdermal and minimally invasive insulin delivery strategies, including relative dose requirements and availability of human data, is summarized in Table 1.

## 7. Needle-Free Jet Injection Systems for Insulin Delivery

Needle-free jet injection systems represent one of the most clinically advanced minimally invasive insulin delivery technologies currently available. Unlike most chemical or nanocarrier-based transdermal strategies, jet injectors have been evaluated in multiple human clinical studies, including randomized and multicenter trials. Given the relative maturity of clinical evidence, this section provides a detailed examination of their historical development, mechanistic basis, pharmacokinetic performance and metabolic outcomes [21].

### 7.1. Historical Development

The concept of needle-free injection dates to the 19th century, with early “aqua-puncture” devices. Modern jet injection technology emerged in the 1930s and 1940s, with early clinical applications in mass vaccination campaigns. However, concerns regarding cross-contamination led to temporary abandonment of these systems in the late 20th century [21].

Contemporary jet injectors differ substantially from early devices. They incorporate single-use cartridges, precise pressure control, and micro-nozzle engineering, significantly improving safety, dosing accuracy, and patient comfort. These advancements have renewed interest in their application for insulin delivery [21].

Given the growing body of clinical evidence, the following subsections provide a detailed analysis of pharmacokinetic optimization, metabolic efficiency, glycemic variability, longitudinal outcomes, and behavioral impact associated with jet injection systems.

### 7.2. Mechanism of Action and Pharmacokinetics

Needle-free jet injectors deliver insulin though a narrow, high-velocity liquid stream that penetrates the skin and disperses insulin within subcutaneous tissue. Unlike conventional needle injections, which deposit insulin in a relatively confined depot, jet injection produces a broader dispersion pattern [21].

This distribution leads to:

○ Faster insulin absorption;
○ Earlier peak plasma insulin concentrations;
○ Pharmacokinetic profiles that more closely resemble endogenous insulin secretion.

Pharmacokinetic studies have quantified these differences. In a randomized crossover study by Engwerda et al. [29], rapid-acting insulin delivered via jet injection reached peak plasma concentration at approximately 31 ± 3 min compared with 64 ± 6 min using conventional pen injection. Additionally, time to maximal glucose infusion rate during euglycemic clamp testing was reduced from approximately 105 min to 51 min, representing nearly a 50% acceleration in metabolic activity. Early area-under-the-curve (AUC 0–60 min) insulin exposure was significantly higher with jet injection, supporting enhanced early bioavailability [29,30].

**Figure 2:**
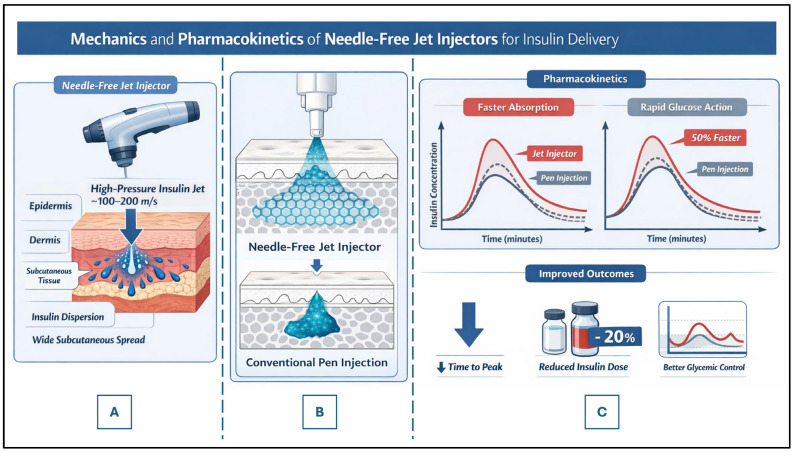
Mechanistic and Pharmacokinetic Comparison Between Needle-Free Injections and Conventional Subcutaneous Pen Injection. (**A**) High-pressure liquid jet delivery (100–200 m/s) penetrates the epidermis and dermis without a solid needle, producing broad dispersion of insulin within the subcutaneous tissue. (**B**) Schematic representation of insulin depot morphology following delivery. Needle-free jet injection generates a wider and more superficial distribution pattern compared with the compact, localized depot formed by conventional pen injection. This expanded surface area facilitates faster capillary uptake. (**C**) Representative pharmacokinetic and pharmacodynamic profiles comparing jet injection (red solid line) with conventional pen injection (blue solid line). The dashed line indicates reference profiles. Jet injection demonstrates earlier peak insulin concentrations (reduced Tmax) and accelerated glucose-lowering action. Arrows denote reductions in time to peak and insulin dose requirements. Clinical studies report approximately 30–50% reductions in time to peak insulin levels and modest reductions in total prandial insulin dose requirements (10–20%) in selected populations.

These properties are particularly relevant for postprandial glucose control and may reduce glycemic variability [21]. A schematic comparison of insulin depot dispersion and pharmacokinetic profiles between conventional subcutaneous injection and needle-free jet injection is presented in Figure 2.

### 7.3. Clinical Evidence of Metabolic Outcomes

The clinical relevance of needle-free insulin delivery systems extends beyond technical feasibility, encompassing pharmacokinetic optimization, metabolic stability, and patient-centered outcomes. Available studies, although heterogeneous in design and population, provide convergent evidence supporting the metabolic non-inferiority (and in some contexts superiority) of needle-free jet injection compared with conventional subcutaneous insulin administration (Table 2) [7,29,31].

#### 7.3.1. Pharmacokinetic Dispersion and Early Postprandial Control

One of the most consistent findings across studies evaluating jet injection systems is the alteration in insulin absorption kinetics. Evidence from both meal test and euglycemic clamp experiments supports this effect [21,29].

In a randomized crossover meal test study by Engwerda et al. [30], rapid-acting insulin administered via needle-free injection achieved earlier plasma insulin peaks and reduced early postprandial hyperglycemia when compared with conventional insulin pens (T-INSmax 51 ± 6 vs. 92 ± 10 min; *p* = 0.003). This acceleration translated into modest reductions in early postprandial glucose excursions during the first 60–90 min, suggesting clinically relevant improvement in early glycemic control [30,34].

Complementary mechanistic evidence from earlier euglycemic clamp studies (Engwerda et al.) [29] demonstrated even more pronounced kinetic differences, with peak insulin concentrations occurring at approximately 31 ± 3 min with jet injection versus 64 ± 6 min with pen injection, and time to maximal glucose infusion rate reduced by nearly 50%. These findings confirm that the acceleration observed in meal-based studies reflects a true pharmacodynamic effect rather than meal-related artifact [29].

Mechanistically, this phenomenon is attributed to the broader and more superficial dispersion of insulin within subcutaneous tissue, which increases the effective absorptive surface area and accelerates capillary uptake [30,34].

From a metabolic standpoint, early postprandial glucose excursions represent a major contributor to glycemic variability and oxidative stress. Therefore, the ability of needle-free delivery to attenuate early hyperglycemia may have implications that extend beyond short-term glucose control, potentially influencing long-term metabolic risk [35].

#### 7.3.2. Insulin Dose Requirements and Basal Metabolic Efficiency

Xing et al. reported that prandial insulin requirements were reduced by approximately 12–18% without deterioration in fasting plasma glucose levels or HbA1c, suggesting improved delivery efficiency rather than simple pharmacodynamic equivalence [28].

The reduced dose requirement may reflect more homogeneous insulin distribution and reduced local pooling, which are known contributors to erratic absorption and insulin resistance at the injection site. From a systems metabolism perspective, this improved efficiency aligns with the concept that delivery mode can influence effective insulin bioavailability independently of molecular formulation [36].

#### 7.3.3. Glycemic Variability as a Metabolic Endpoint

Beyond mean glucose values and HbA1c, several studies incorporated continuous glucose monitoring to assess glycemic variability. Sun et al. [33] observed a statistically significant reduction in coefficient of variation (CV%) of fasting glucose levels, decreasing from approximately 28% with conventional injection to 22–24% with jet injection during continuous glucose monitoring. Glycemic variability has emerged as an independent predictor of endothelial dysfunction, inflammation, and microvascular complications, underscoring its relevance as a metabolic endpoint [33,37].

The observed reduction in variability supports the hypothesis that needle-free jet injection produces a more predictable and reproducible insulin absorption profile. Importantly, this benefit cannot be captured solely by HbA1c measurements, highlighting the added value of integrating advanced glucose metrics when evaluating novel delivery technologies [33,34,38,39].

#### 7.3.4. Longitudinal Glycemic Outcomes and HbA1c Reduction

In the 16-week multicenter trial by Ji et al. [40], HbA1c reduction was approximately 0.4–0.6% greater in the jet injection group compared with insulin pen users, without increased hypoglycemia incidence. While the absolute difference may appear modest, even small HbA1c reductions are associated with meaningful decreases in microvascular risk at the population level [31,40].

Notably, this improvement occurred without an increase in hypoglycemia, suggesting that enhanced metabolic control was achieved through improved pharmacokinetic alignment rather than aggressive dose escalation. This observation reinforces the concept that delivery technology can modulate metabolic outcomes independently of insulin formulation [31,40].

#### 7.3.5. Psychological Insulin Resistance and Adherence as Metabolic Modifiers

Wang et al. [5] uniquely addressed psychological insulin resistance, adherence, and patient-reported outcomes in addition to glycemic endpoints. Their findings indicate that needle-free injection significantly improves patient attitudes toward insulin therapy, reduces treatment-related anxiety, and enhances adherence [5].

From a metabolic systems perspective, adherence functions as a behavioral modifier of metabolic control. Even optimal pharmacological regimens fail in the context of inconsistent administration. Therefore, improvements in adherence mediated by reduced pain and fear may indirectly contribute to the superior glycemic outcomes observed in some studies [5,6,41].

This behavioral–metabolic interaction is particularly relevant in type 2 diabetes, where delayed insulin initiation and suboptimal adherence are major barriers to sustained metabolic control [18].

#### 7.3.6. Methodological Limitations and Interpretive Caution

Despite encouraging results, several limitations must be acknowledged. Many studies employ crossover designs with relatively short follow-up periods, limiting assessment of long-term metabolic outcomes and safety. Sample sizes are often modest, and heterogeneity in insulin regimens, patient populations, and monitoring methods complicates direct comparison [5,28,30,31,33].

Additionally, most studies have been conducted in controlled research settings, which may not fully capture real-world variability in device handling, injection technique, and patient behavior. These factors highlight the need for pragmatic trials and long-term observational studies incorporating metabolic, dermatological, and behavioral endpoints [5,28,30,31,33].

#### 7.3.7. Integrated Metabolic Interpretation

Taken together, existing evidence suggests that needle-free insulin delivery systems influence metabolic control through three interrelated mechanisms:

(a) Pharmacokinetic optimization, resulting in faster and more uniform insulin absorption.
(b) Reduction in glycemic variability, as captured by continuous glucose monitoring metrics.
(c) Improved adherence and treatment acceptance, mitigating behavioral barriers to effective therapy.

These mechanisms operate synergistically, positioning needle-free delivery as more than a technological convenience—it represents a potential metabolic intervention capable of modulating both biological and behavioral determinants of glycemic control [5,28,30,31,33,34,36,40,41,42].

## 8. Impact on Treatment Adherence and Patient-Reported Outcomes

Beyond pharmacokinetics, needle-free insulin delivery directly addresses one of the most persistent barriers in diabetes management: psychological insulin resistance. Fear of needles, anticipation of pain, and negative emotional associations with injections are major contributors to poor adherence [6].

Studies consistently report:

- Reduced pain perception with jet injectors;
- Improved treatment satisfaction;
- Lower scores on insulin resistance and negative attitude scales;
- Higher self-reported adherence.

In controlled crossover trials, visual analog scale (VAS) pain scores have been reported to decrease by approximately 30–50% compared with conventional needle injection, while treatment satisfaction scores increased significantly in patient-reported outcome measures [5,21].

Improved adherence is not merely a behavioral outcome, it translates into tangible metabolic benefits, including improved glycemic stability and reduced long-term complication risk [6,41].

Importantly, adherence acts as a biological amplifier of pharmacokinetic optimization. Even modest improvements in insulin absorption kinetics may produce clinically meaningful glycemic benefits when combined with increased treatment consistency. Thus, behavioral and metabolic effects should be interpreted as synergistic rather than independent phenomena.

## 9. Integration with Continuous Glucose Monitoring

The increasing use of continuous and flash glucose monitoring systems has transformed the assessment of insulin therapy. Metrics such as time in range, glycemic variability, and time below range provide a more nuanced view of metabolic control than HbA1c alone [10,38,43,44,45,46,47,48,49,50].

Needle-free insulin delivery appears particularly well suited for integration with these technologies, as its rapid and consistent absorption profile aligns with dynamic glucose monitoring. Studies using continuous glucose monitoring have demonstrated reduced variability and improved early glycemic responses when insulin is delivered via jet injection [32,33].

Specifically, reductions in fasting glucose coefficient of variation of approximately 4–6% have been observed in crossover studies, accompanied by trends toward improved early postprandial time-in-range during the first 2–3 h following meals. Although absolute TIR differences remain modest in short-term studies, these findings suggest improved absorption predictability [32,33].

Because glycemic variability has been independently associated with oxidative stress, endothelial dysfunction, and microvascular complications, improvements in early glucose stability may carry implications beyond mean HbA1c reduction [50].

## 10. Safety Considerations and Limitations of Transdermal and Needle-Free Insulin Delivery

Safety evaluation of alternative insulin delivery platforms must consider both acute and long-term implications, including dermatologic, immunologic, and system effects [41].

Transdermal chemical enhancement systems (such as ionic liquid-based patches and nanovesicle formulations) introduce excipients designed to disrupt or modulate stratum corneum lipid organization. Although recent studies report minimal acute irritation and favorable biocompatibility in animal models, chronic exposure data remain limited. Repeated lipid fluidization or polymer–lipid interactions may theoretically alter barrier integrity over time, particularly in individuals with pre-existing dermatologic conditions [11,20].

Despite the promising metabolic and behavioral benefits of transdermal and needle-free insulin delivery systems, several safety considerations and limitations must be acknowledged. From a dermatological perspective, repeated skin exposure to mechanical or microstructural disruption raises concerns regarding local reactions, including erythema, bruising, transient edema, and, less frequently, superficial hematomas. Available clinical data indicate that these reactions are generally mild and self-limited with no significant increase in infection rates when modern single-use systems are employed [7,8,21].

Reported rates of mild local erythema in microneedle feasibility studies range between 5 and 15%, typically resolving within 24–48 h without intervention. Similarly, jet injection-related bruising has been reported in fewer than 10% of users in most controlled studies [29,30,32,34,35,36,42].

In the case of polyzwitterion-mediated systems, no histologic evidence of inflammatory infiltration or epidermal damage was observed in short-term animal models. However, long-term immunogenicity of conjugated insulin formulations has not yet been established in human populations [11].

Microneedle systems generally demonstrate favorable tolerability profiles, with minimal erythema and rapid microchannel closure. Nonetheless, concerns persist regarding sterility, potential infection risk in immunocompromised patients, and mechanical reliability of dissolvable polymers. The presence of microscopic punctures, while minimally invasive, still represents a barrier breach [12,15,22].

Needle-free jet injectors present a distinct safety profile. Earlier generations of devices were associated with cross-contamination risks during mass vaccination campaigns. However, contemporary systems incorporate disposable cartridges, single-patient use, and precise pressure regulation, substantially mitigating these risks. Current clinical studies have not demonstrated increased rates of local or systemic adverse events compared with conventional subcutaneous injection [7,8,21].

Another limitation relates to dose precision and reproducibility. While modern jet injectors offer improved control over delivery pressure and volume, variability in skin thickness, hydration, and tissue composition may influence insulin dispersion and absorption. Similarly, microneedle-based systems face challenges related to consistent dose loading, mechanical integrity, and insulin stability within polymeric matrices [7,8,21,29,36].

From a clinical standpoint, long-term safety data remain limited. Most available studies are short- to medium-term and focus on glycemic outcomes rather than chronic dermatological or immunological effects. Large-scale, long-duration trials are therefore required to establish the safety profile of these technologies across diverse patient populations, including older adults and individuals with comorbid skin conditions [5,28,30,31,33].

## 11. Translational and Regulatory Challenges

Despite promising mechanistic advances, translation from experimental models to routine clinical use requires overcoming substantial logistical and regulatory barriers.

Chemical permeation systems and polymer-conjugated insulin formulations represent combination products that integrate drug and device components. Regulatory agencies require robust evidence of bioequivalence, reproducibility, chronic safety, and stability under real-world storage conditions. Additionally, supraphysiologic dosing requirements observed in many preclinical studies raise concerns regarding cost-effectiveness and large-scale insulin supply sustainability [11,20].

The translation of transdermal and needle-free insulin delivery systems from experimental or niche clinical use to widespread adoption involves several non-biological challenges. Manufacturing scalability, cost-effectiveness, and regulatory approval pathways represent critical hurdles [7].

Microneedle systems, in particular, face challenges related to large-scale production, sterility assurance, and long-term stability of biologically active insulin. Regulatory agencies require robust evidence of consistency, safety, and efficacy, which has slowed clinical translation despite promising preclinical data [15,22].

Needle-free jet injectors are closer to clinical integration, yet their adoption is influenced by device cost, training requirements, and reimbursement policies. Healthcare providers must be trained not only in device operation but also in patient education to ensure proper use and realistic expectations. In resource-limited settings, the initial investment required for device acquisition may limit accessibility, despite potential long-term benefits through improved adherence and metabolic control [14].

From a regulatory perspective, harmonization of standards for needle-free delivery systems is still evolving. Clear guidance on bioequivalence, pharmacokinetic assessment, and post-marketing surveillance will be essential to support broader implementation. Importantly, although jet injection systems use approved insulin formulations without chemical modification, them or nanocarrier-based systems, may require full evaluation as new drug–device combination products.

## 12. Future Perspectives: Toward Patient-Centered and Metabolically Optimized Insulin Therapy

The future of insulin therapy is increasingly defined by integration with digital health platforms, continuous glucose monitoring (CGM), and decision-support algorithms. Delivery technologies that offer faster, more predictable absorption kinetics may enhance the effectiveness of hybrid closed-loop systems.

Transdermal sustained-delivery platforms may theoretically serve basal insulin needs, whereas rapid-dispersion systems such as jet injectors may optimize prandial control. Rather than competing modalities, these technologies could function complementarily within precision-based therapeutic strategies.

Moreover, minimally invasive delivery methods may reduce psychological resistance to insulin use and facilitate earlier treatment intensification in type 2 diabetes. Early glycemic control is strongly associated with long-term reduction in microvascular and macrovascular complications, suggesting that improved delivery systems could exert downstream epidemiological impact.

Advances in materials science, nanotechnology, and bioresponsive polymers may ultimately enable hybrid platforms combining glucose sensing, automated insulin release, and painless delivery—approaching a fully patient-friendly closed-loop paradigm.

Future comparative trials should incorporate time-in-range, glycemic variability, patient-reported outcomes, and cost-effectiveness analyses to determine whether delivery modality can independently influence long-term metabolic risk.

## 13. Conclusions

Transdermal and needle-free insulin delivery technologies represent a meaningful evolution in diabetes management. Emerging chemical permeation strategies demonstrate sophisticated mechanistic approaches to overcoming the skin barrier, including lipid fluidization, polyzwitterion-mediated transport, and nanovesicle encapsulation. These systems achieve measurable pharmacodynamic effects but often require supraphysiologic dosing, raising questions regarding scalability and long-term feasibility.

Microneedle platforms provide a mechanically assisted compromise, enabling near-physiologic dosing with reduced pain, yet face challenges in payload capacity and manufacturing standardization.

Needle-free injectors, in contrast, modify delivery mechanics without altering insulin chemistry. They demonstrate improved pharmacokinetic profiles, faster glucose correction, and preserved dose flexibility, positioning them as immediately translatable technologies within existing clinical infrastructures.

Ultimately, the evolution of insulin delivery must balance mechanistic innovation with clinical practicality. Technologies that reduce treatment burden while maintaining pharmacologic fidelity and scalability are most likely to achieve durable impact.

In this context, transdermal and needle-free systems should not be viewed merely as alternative administration routes but as integral components of a patient-centered and metabolically optimized therapeutic strategy.

## Figures and Tables

**Table 1:** Transdermal and Needle-Free Insulin Delivery Strategies: Mechanisms, Dose Requirements, and Translational Considerations.

Strategy	Mechanism	Dose vs. SC	Human Data	Key Limitation	Ref.
Chemical enhancers	Lipid disruption of stratum corneum	>5–10× SC	No (animal)	Skin irritation, low efficiency	[24]
Iontophoresis	Electrical driving force	Variable	Limited	Device complexity	[25]
Electroporation	Transient aqueous pores	Variable	Limited	Tissue damage risk	[26]
Ultrasound	Cavitation/hyperthermia	Variable	Limited	Inconsistent dosing	[27]
Microneedles	Microchannel bypass	≈SC dose	Yes (pilot)	Payload limit	[15]
Jet injection	High-pressure dispersion	≈SC or <10–20%	Yes	Device cost	[28]
IL/O systems	Lipid fluidization	5× SC	No (animal)	High dose requirement	[20]
Polyzwitterion	Polymer-mediated SC traversal	~20× SC	No (animal)	Supraphysiologic dosing	[11]
Ethosomes	Lipid nanovesicles	3× SC	No (animal)	Dose escalation	[20]

Comparative overview of transdermal and needle-free insulin delivery strategies, summarizing their principal mechanisms of action, relative insulin dose requirements versus conventional subcutaneous (SC) injection, extent of available human clinical evidence, and key translational limitations. Reported dose ratios reflect representative values derived from published experimental or clinical studies and highlight the efficiency gap observed in several chemical permeation systems. Notably, among platforms, microneedle-based systems and needle-free jet injectors are the only approaches evaluated in human clinical settings to date, whereas most chemical and nanocarrier-based strategies remain confined to preclinical models. Dose ratios represent nominal insulin amounts administered in the referenced studies and should not be interpreted as direct pharmacokinetic equivalence. In several cases, particularly preclinical animal models, higher loaded doses were required to achieve comparable glycemic effects due to limited transdermal bioavailability. SC: subcutaneous; IL/O: ionic liquid-in-oil.

**Table 2:** Clinical studies evaluating the efficacy, pharmacokinetics, and patient-reported outcomes of needle-free jet insulin injection compared with conventional pen injection.

Study	Population	Design	Comparator	Duration	Key Quantitative Outcome	Key Findings
Engwerda et al. (2013) [30]	T1D & T2D (*n* = 24)	Randomized crossover	Insulin pen	Acute (6 h meal test)	T-INSmax: 51 vs. 92 min (*p* = 0.003)	Faster insulin absorption, modest reduction in early (0–60 min) postprandial hyperglycemia
Xing et al. (2019) [28]	T2D (*n* = 62)	Randomized crossover	Insulin pen	7–14 days per cycle	Insulin dose for FBG: 16.14 ± 5.13 vs. 19.25 ± 6.2 U/d (NFII vs. CIP), (*p* = 0.0046)	Comparable glycemic control with lower insulin requirement
Sun et al. (2020) [32]	T2D (*n* = 26)	Self-comparative observational	Conventional injection	14 days	24 h mean glucose: 8.3 ± 1.5 vs. 8.8 ± 1.5 mmol/L (jet vs. pen); PPG lunch: 9.9 ± 3.0 vs. 11.9 ± 2.8.	Lower 24 h mean glucose and reduced postprandial excursions; no significant difference in overall variability
Ji et al. (2020) [31]	T2D (*n* = 412)	Multicenter RCT	Insulin pen	16 weeks	ΔHbA1c −0.4 to −0.6% vs. pen	Superior HbA1c reduction; non-inferior safety
Sun et al. (2022) [33]	T2D (*n* = 43)	Crossover	Conventional injection	28 days	CV-FBG: 8.92 [4.73–12.46]% vs. 8.1 [5.84–13.29]%.	Reduced fasting glucose variability via CGM
Wang et al. (2024) [5]	T2D (*n* = 64)	Crossover	Insulin pen	5–6 weeks	ITASS 53.7 ± 12.7 vs. 58.9 ± 12.3; ITAQS 46.3 ± 10.9 vs. 43.8 ± 12; ITSQS 66.6 ± 10.2 vs. 62.4 ± 10.0 (NFII vs. CIP)	Improved treatment satisfaction and reduced psychological resistance

Data summarize randomized crossover and parallel-group studies in type 1 and type 2 diabetes. Reported outcomes include pharmacokinetic parameters (T-INSmax), glycemic variability indices derived from continuous glucose monitoring (coefficient of variation [CV], mean amplitude of glycemic excursions [MAGE]), glycated hemoglobin (HbA1c) changes, insulin dose requirements, and patient-reported satisfaction metrics. Collectively, these studies suggest accelerated insulin absorption, modest improvements in early postprandial glycemia, reduced insulin dose requirements in selected populations, and improved treatment acceptance without compromising safety.

## Data Availability

No new data were created or analyzed in this study. Data sharing is not applicable to this article.

## Abbreviations

The following abbreviations are used in this manuscript:

T1D	Type 1 diabetes
T2D	Type 2 diabetes
HbA1c	Hemoglobin glycated
RCT	Randomized controlled trial
CGM	Continuous glucose monitoring
AUC	Area under curve
SC	Subcutaneous
IL/O	Ionic liquid-in-oil
T-INSmax	Time to maximum plasma concentration
FBG	Fasting blood glucose
PPG	Postprandial glucose
CV	Coefficient of variation
ITASS	Insulin treatment appraisal scale—satisfaction subscale
ITAQS	Insulin therapy appraisal questionnaire—satisfaction
ITSQS	Insulin treatment satisfaction questionnaire scale
NFII	Needle-Free insulin injection
CIP	Conventional insulin pen
